# Hydrocolloids of Egg White and Gelatin as a Platform for Hydrogel-Based Tissue Engineering

**DOI:** 10.3390/gels9060505

**Published:** 2023-06-20

**Authors:** Karinna Georgiana Pele, Hippolyte Amaveda, Mario Mora, Carlos Marcuello, Anabel Lostao, Pilar Alamán-Díez, Salvador Pérez-Huertas, María Ángeles Pérez, José Manuel García-Aznar, Elena García-Gareta

**Affiliations:** 1 Multiscale in Mechanical & Biological Engineering Research Group, Aragon Institute of Engineering Research (I3A), School of Engineering & Architecture, University of Zaragoza, 50018 Zaragoza, Aragon, Spain; pelekarinna@gmail.com (K.G.P.); alamanp@unizar.es (P.A.-D.); angeles@unizar.es (M.Á.P.); jmgaraz@unizar.es (J.M.G.-A.); 2 Instituto de Nanociencia y Materiales de Aragón (INMA), CSIC and University of Zaragoza, 50018 Zaragoza, Aragon, Spain; hippo@unizar.es (H.A.); mmora@unizar.es (M.M.); cmarcuel@unizar.es (C.M.); aglostao@unizar.es (A.L.); 3 Laboratorio de Microscopías Avanzadas (LMA), University of Zaragoza, 50018 Zaragoza, Aragon, Spain; 4 Fundación ARAID, 50018 Zaragoza, Aragon, Spain; 5 Department of Chemical Engineering, Faculty of Sciences, University of Granada, 18071 Granada, Andalusia, Spain; shuertas@ujaen.es; 6 Aragon Institute for Health Research (IIS Aragon), Miguel Servet University Hospital, 50009 Zaragoza, Aragon, Spain; 7 Division of Biomaterials & Tissue Engineering, UCL Eastman Dental Institute, University College London, London NW3 2PF, UK

**Keywords:** hydrocolloids, egg white, gelatin, hydrogels, tissue engineering, microfluidics

## Abstract

Innovative materials are needed to produce scaffolds for various tissue engineering and regenerative medicine (TERM) applications, including tissue models. Materials derived from natural sources that offer low production costs, easy availability, and high bioactivity are highly preferred. Chicken egg white (EW) is an overlooked protein-based material. Whilst its combination with the biopolymer gelatin has been investigated in the food technology industry, mixed hydrocolloids of EW and gelatin have not been reported in TERM. This paper investigates these hydrocolloids as a suitable platform for hydrogel-based tissue engineering, including 2D coating films, miniaturized 3D hydrogels in microfluidic devices, and 3D hydrogel scaffolds. Rheological assessment of the hydrocolloid solutions suggested that temperature and EW concentration can be used to fine-tune the viscosity of the ensuing gels. Fabricated thin 2D hydrocolloid films presented globular nano-topography and in vitro cell work showed that the mixed hydrocolloids had increased cell growth compared with EW films. Results showed that hydrocolloids of EW and gelatin can be used for creating a 3D hydrogel environment for cell studies inside microfluidic devices. Finally, 3D hydrogel scaffolds were fabricated by sequential temperature-dependent gelation followed by chemical cross-linking of the polymeric network of the hydrogel for added mechanical strength and stability. These 3D hydrogel scaffolds displayed pores, lamellae, globular nano-topography, tunable mechanical properties, high affinity for water, and cell proliferation and penetration properties. In conclusion, the large range of properties and characteristics of these materials provide a strong potential for a large variety of TERM applications, including cancer models, organoid growth, compatibility with bioprinting, or implantable devices.

## 1. Introduction

Tissue engineering and regenerative medicine (TERM) emerged with the original purpose of producing tissues and organs in the laboratory ready for clinical implantation [1,2]. Only three components are needed to achieve this monumental task: cells, molecular/environmental cues, and a biomaterial that acts as a scaffold for the newly built tissue or organ [1]. In the last decade, this original purpose has evolved towards the production of tissue analogues and models to study organ formation, tumor progression, or new drug therapies, among others [2,3,4,5]. These models can be 2D or 3D, including intelligent cell culture surfaces, organs-on-chip, organoids, or scaffold-based models [6,7,8,9,10,11]. One component remains key in their development: a biomaterial scaffold. New and innovative materials are needed for producing these scaffolds, especially those derived from natural sources that offer low production costs, easy availability, and high bioactivity [12]. One such material is chicken egg white (EW), which is a disregarded protein-based material with potential to be used in different applications [12,13].

EW is a native biomaterial with compelling structural, biological, and physico-chemical properties. Temperature can modulate its viscosity and its transparency is fitting for 3D culture systems [14]. Importantly, EW is suitable for various biomedical applications due to its antibacterial, antihypertensive, anti-inflammatory, healing-enhancing, and cell growth stimulatory features [15]. To name a limitation, as with any biomaterials of natural origin, batch-to-batch variation might occur because of chicken age, feeding, or egg storage time [12]. A prominent example is collagen type I, which presents significant batch-to-batch variation even when obtained from the same provider [2]. Collagen is the most used protein-based biomaterial in TERM models and implants [2], thereby demonstrating the enormous potential of natural materials for TERM applications.

**Figure 1:**
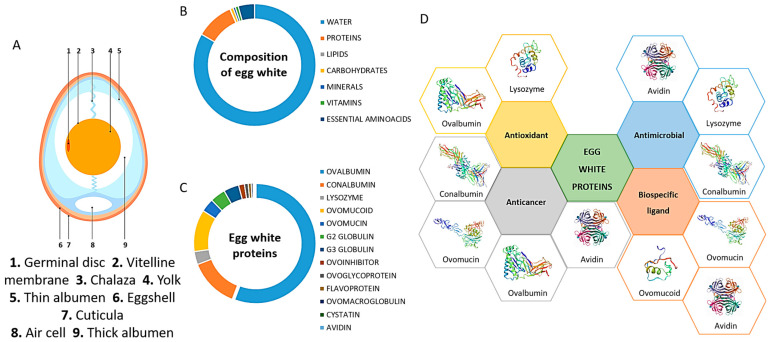
(**A**) Major structural components of chicken egg. (**B**) Composition of egg white showing the relative abundance of the different components. (**C**) Relative abundance of proteins present in egg white. (**D**) Biological activities of the main proteins found in egg white.

The major structural components of chicken egg are shell, shell membrane, yolk, and white (Figure 1A). Specifically, EW is found between the eggshell membrane layers and the yolk [12]. Eggshell is composed of calcium and phosphate and is porous to allow air permeation to the interior. Eggshell membrane, located between the eggshell and EW, has a protein-based structure and supports the formation of proteins and enzymes. It has three morphologically distinct layers, namely outer, inner, and limiting membranes, that protect the egg content from bacteria. Egg yolk is found suspended in the EW via the chalaza, two connection tissues, and is a source of nutrients and vitamins for the embryo, as well as a reservoir of immunoglobulin. EW functions as a second protection layer to prevent bacterial infections from reaching the yolk [12].

In terms of composition, EW is a hydrocolloid comprising a mixture of water (~85%), proteins (~10%), carbohydrates, minerals, vitamins, and essential aminoacids [16,17] (Figure 1B). Proteins are the main element of EW, contributing to its physical and biological properties, which makes them ideal bioactive compounds for medical, pharmaceutical, and bioengineering applications [15,17] (Figure 1C,D). They are globular and classified into two groups according to their abundance into main (>83%) and minor proteins (<17%) [12]. EW’s high viscosity, which decreases with increasing temperature, is due to the high content of ovomucin. The emulsified hydrocolloid’s stability, as well as proteins’ emulsifying activity, depends on pH, salt presence, and protein concentration [18].

Gelatin is a natural biopolymer obtained through the denaturation and partial hydrolysis of collagen in the connective tissue of muscles, skin, and bones of animals [19]. Gelatin is widely used in biomedical science, and pharmaceutical and food industries because of its biocompatibility, bioactivity, biodegradability, non-antigenicity, plasticity, adhesiveness, and unique gel properties [19]. Hydrogen and electrostatic bonds, as well as hydrophobic interactions, stabilize gelatin hydrogels [19], which are thermally reversible and melt around human body temperature, giving them melt-in-mouth properties. This gelling agent is used for various biological and functional motives in biomedical, pharmaceutical, cosmetic, and food formulations [19,20].

Gelation involves the formation of a continuous 3D network by chemical or physical crosslinking that traps and immobilizes the liquid within it to yield a polymeric structure of infinite viscosity, i.e., a gel that resists flow under pressure. Hydrogels are formed when the trapped liquid is water. Hydrogels can be described as hydrophilic 3D porous cross-linked polymeric networks, which are capable of absorbing and retaining large amounts of water, including aqueous biological fluids, without fracturing their 3D structure [21]. Hydrogels hold great potential for TERM as they mimic the natural extracellular matrix (ECM) due to their hydrated nature, viscoelastic properties, and ability to incorporate cells or growth factors [22,23].

EW is denatured by heating to yield an ordered and irreversible gel network structure that is formed by hydrophobic interactions [12]. This functional property of EW to gel plays an important role in products derived from the food industry, e.g., dairy products, jelly, sausage, and gel products [20]. Gelatin exhibits thermoreversible properties, i.e., below the sol-gel transition temperature, triple helix from spiral molecules is formed, structuring into an elastic hydrogel; while in hot water, hydrogen and electrostatic bonds are decomposed, soluble collagen is denatured, and the molecules produce a hydrocolloid [19]. When gelatin is mixed with other compounds at certain concentrations, the resulting mixtures also show thermoreversible properties [20]. As just seen, the gel properties of EW and gelatin have been studied and reported however, the gel properties of mixed EW and gelatin hydrocolloids have not received nearly the same attention. Indeed, mixtures of EW and gelatin have been investigated in the food industry [20,24,25]. For instance, edible hydrogels of EW were created using gelatin as a porogen and texture modifier, thereby obtaining highly porous hydrogels after gelatin depletion [24]. Pérez-Huertas and colleagues investigated EW albumin/gelatin dispersion gelled onto cold plasma-activated glass, where the increased roughness of the gel surface can increase gel decomposition by the gastrointestinal enzymes as well as change adsorption of active ingredients on the gel surface [25]. However, to the best of our knowledge, mixtures of EW and gelatin have not been reported for TERM applications. Indeed, there are very few reports on the use of EW for tissue engineering, which has been mixed with synthetic polymers [26,27] or ceramics [28]. A relevant study by Carpena and colleagues reported 3D porous sponges of ovomucin and gelatin, where the latter was used to stabilize the foams that rendered the sponges [29].

**Figure 2:**
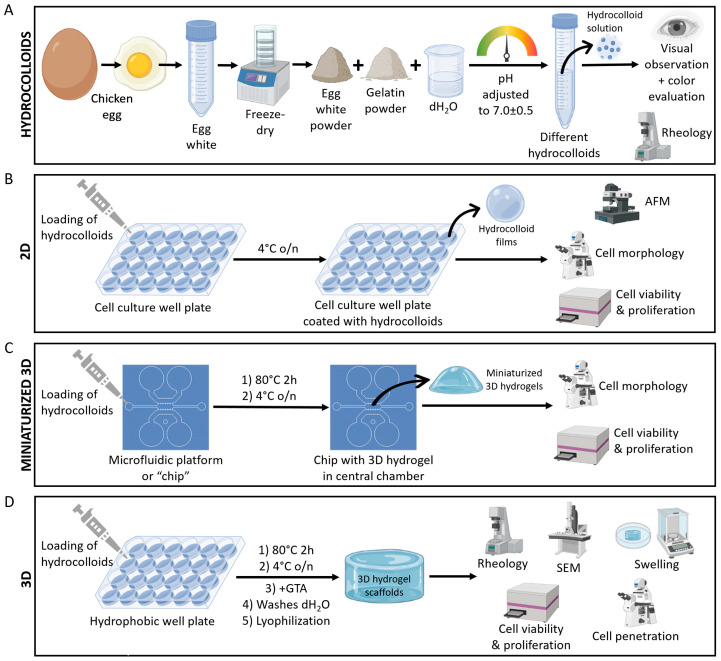
Visual summary of the experimental work carried out in this study. (**A**) Hydrocolloid solutions of EW and gelatin. (**B**) 2D hydrocolloid films. (**C**) Miniaturized 3D hydrogels of EW and gelatin inside microfluidic platforms. (**D**) 3D hydrogel scaffolds of EW and gelatin. AFM: atomic force microscopy; SEM: scanning electron microscopy; o/n: overnight; and GTA: glutaraldehyde. A detailed experimental design summary can be found in Section 4.1.

Taking inspiration from the food chemistry and technology field, we aimed to investigate hydrocolloids of both EW and gelatin as a suitable platform for hydrogel-based tissue engineering. We hypothesized that the temperature-dependent gelation properties of both materials can be used to create efficient 2D coating films, miniaturized 3D hydrogels in microfluidic devices, or to fabricate 3D hydrogel scaffolds. To address both the aim and hypothesis of this study, our experimental design consisted of four differentiated blocks, visually summarized in Figure 2.

## 2. Results and Discussion

The development and fabrication of EW and gelatin hydrogel-based structures, as well as the study of their material and cell interaction properties, are an important first step in biomaterial-based tissue engineering applications of these hydrocolloid mixtures, which have already been investigated for food technology purposes [20,24,25].

### 2.1. Egg White and Gelatin Hydrocolloids: Appearance and Rheology

**Figure 3:**
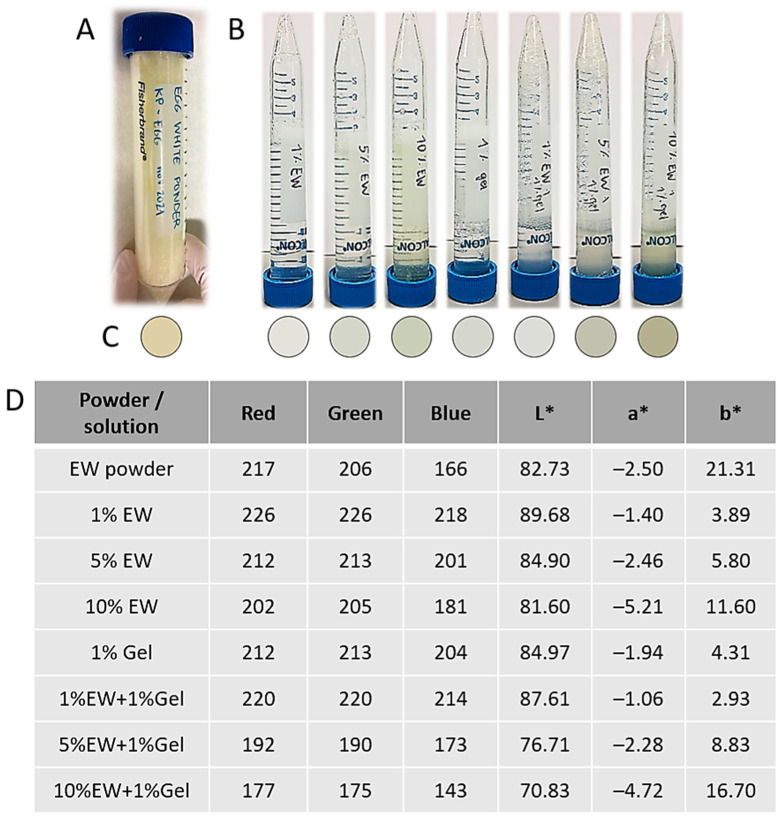
(**A**) Macroscopic appearance of lyophilized EW powder. (**B**) Macroscopic appearance of the different hydrocolloid solutions. (**C**) Color of the different hydrocolloid solutions. (**D**) Color evaluation using the RGB (red, green, blue) and L*a*b* systems.

To create hydrocolloid-based coatings and scaffolds, EW was lyophilized into a powder, which was yellowish in color (Figure 3A). Obtaining a powder allowed us to control the final EW concentration in the hydrocolloids (Table 1). This EW powder was mixed with gelatin and water in different proportions to obtain the colloidal solutions outlined in Table 1. These solutions were all translucid, yellowish in color, and more viscous with higher EW content (Table 1 and Figure 3B–D). Translucidity is an intrinsic property of colloidal solutions because of the particle size dispersed in the solvent [30]. Finally, the addition of EW alkalinized the solution, which had to be neutralized for cellular compatibility.

**Figure 4:**
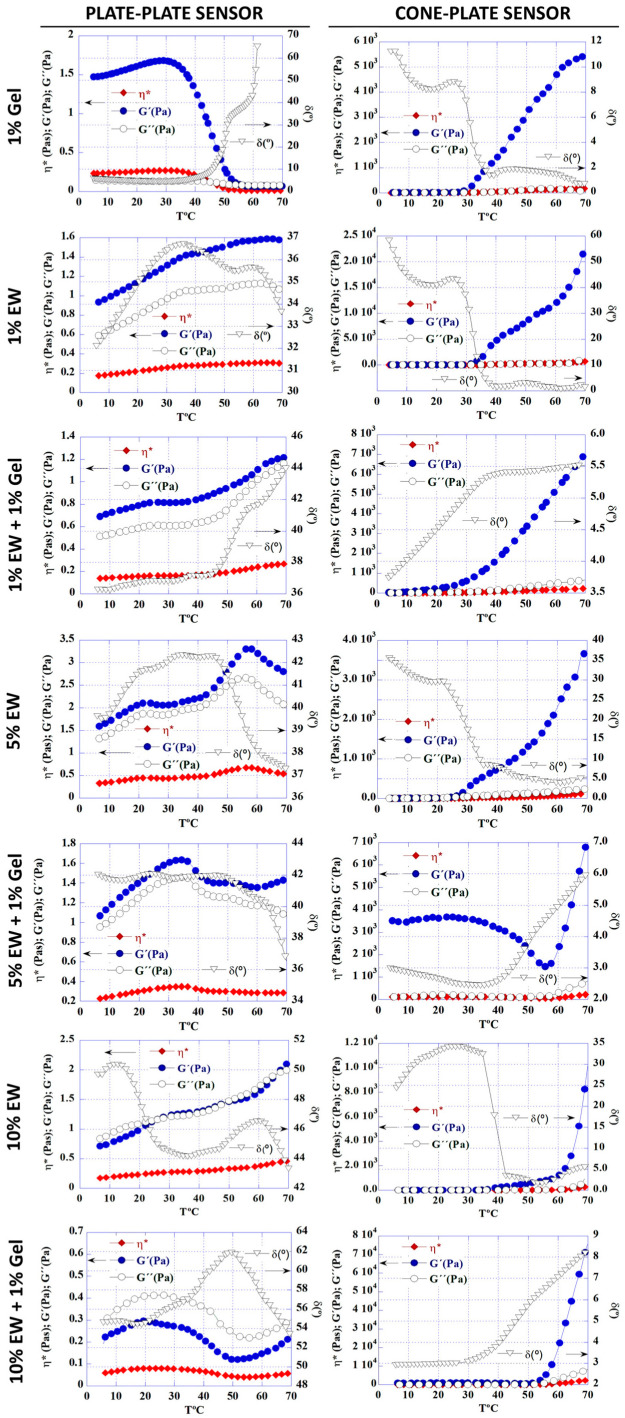
Rheological analysis of the hydrocolloid solutions using a plate-plate sensor (**graphs on the left**) or a cone-plate one (**graphs on the right**). Graphs show the storage G’ and loss modulus G” (blue and green respectively, measured in Pa), phase angle δ (black, measured in °), and specific viscosity η* (red, measured in Pa.s) of each sample recorded against increasing temperature (T, measured in °C).

Rheological analysis of the different hydrocolloids was carried out with two different sensor geometries, namely plate-plate and cone-plate. The evolution between solution and gel states was followed by increasing temperature from 5 to 70 °C at a constant heating rate [31,32]. The choice of sensor had a clear effect on the results, most noticeably for the 1% gelatin solution (Figure 4). With the plate-plate sensor it was observed that the storage modulus G’, loss modulus G”, and specific viscosity η* decreased as the temperature increased, whilst the phase angle δ increased, indicating that the 1% gelatin solution transitioned from a gel to a liquid with increasing temperature, a behavior widely reported in the literature [19,33,34]. However, with the cone-plate sensor, the opposite behavior was observed, with the solution becoming a liquid as the temperature increased.

For the EW solutions, there was a sol-gel transition as the temperature was increased, which was very clear when a cone-plate sensor was used. Overall, the mixed hydrocolloids showed an increased viscosity and gel behavior over the individual hydrocolloids. For some hydrocolloids, i.e., 5% EW, 5% EW + 1% Gel, and 10% EW + 1% Gel, an interesting behavior was observed: the G’ was almost constant until a slight decrease was observed, followed by an increment in G’, which actually was observed twice for the 5% EW solution with the plate-plate sensor. This would indicate that the nature of these solutions is mostly a gel until a destabilization of the system is followed by a rearrangement into a more viscous gel, suggesting a transient and dynamic system [35,36]. Rheological analysis showed a predominantly elastic behavior over a viscous one as the modulus of storage G’ was higher than the loss modulus G”. The exception was the 10% EW + 1% Gel sample that, when measured with the plate-plate sensor, showed a higher G” than G’. Due to the difference in behavior with increasing temperature between the hydrocolloids, results suggested that temperature and EW concentration can be used to fine-tune the viscosity of the resulting gels.

Regarding the disparity in results between the two sensors used, selecting the optimal sensor geometry is a prerequisite for reliable rheological measurements, as each geometry has limitations [37,38]. Whilst the cone-plate geometry is suitable for most samples, for rheological measurements in a temperature range, like in this study, the thermal expansion of the sample and measuring system is less noticeable in large gaps. Therefore, the use of a cone-plate sensor may be limited in these situations [37], as our results for the 1% gelatin solution suggest. Moreover, in a cone-plate sensor, the shear rate is uniform throughout the sample, whilst in a plate-plate sensor it varies with the plate radius, which may have an effect on results [39]. Finally, given the characteristics of the samples prepared in this study, as well as the difference in sample volume necessary for measurement (500 μL for the cone-plate sensor versus 1000 μL for the plate-plate sensor), sample drifting may occur during measurement with the cone-plate sensor. With the plate-plate sensor, the plate surface is sufficient to allow measurement in the absence of sample drifting, thereby adequately transmitting strains. Other scientists have also pointed out the importance of choosing the right sensor for rheological measurements, since they found differences in results related to gel behavior [38,39], thereby suggesting the complexity of colloidal and gel systems and the importance of their thorough characterization.

### 2.2. Two-Dimensional Hydrocolloid Films of Egg White and Gelatin: Topography

Tissue engineering does not only concern 3D applications, but also 2D ones. Examples are cell sheets, intelligent and optimized cell culture surfaces, or functional coatings [7,40,41,42,43,44,45]. Cell sheet tissue engineering involves the preparation, harvesting, manipulation, and transplantation of cell sheets that conserve cell-to-cell binding and adhesive proteins on the basal side [7]. A cell culture material that allows cell sheet formation and subsequent detachment by simply changing temperature is typically used [7]. Much research on 2D tissue engineering focuses on the investigation of optimized cell culture surfaces that promote cell growth and allow investigation of cellular phenomena in response to stimuli [11,46,47,48]. Finally, functional coatings are important to, for example, enhance osseointegration of metallic implants [41,43].

**Figure 5:**
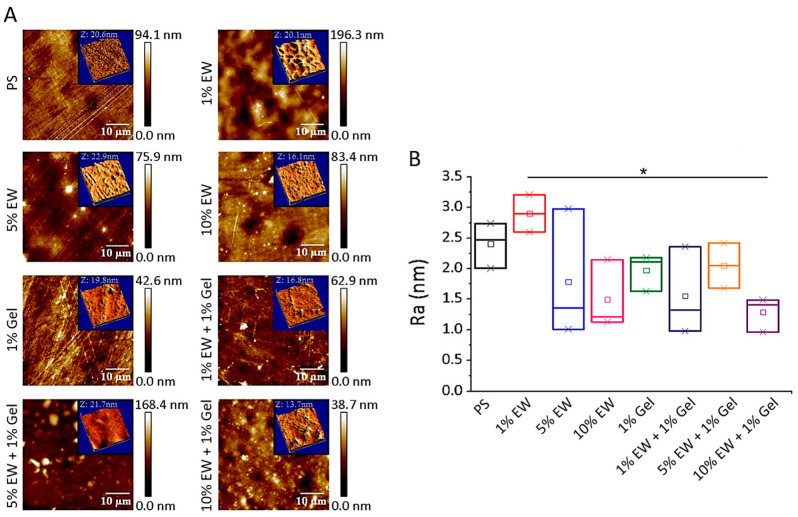
(**A**) Representative 2D topography AFM images of the different hydrocolloid films. Scan size is 50 µm × 50 µm. The inset panels represent 3D topography maps of 3 µm × 3 µm areas. (**B**) Box charts represent the mean values of the roughness average (Ra) parameter for the 3 tested surface areas (N = 3) for each analyzed sample. * *p* < 0.05.

We tested our hydrocolloids as cell culture films and characterized their topography at the nanoscale by AFM (Figure 5), as the literature shows that it may affect cell behavior [11,48]. Our results showed that all surfaces were quite flat with calculated mean roughness arithmetic average (Ra) values below 3 nm (Figure 5B). We used a cell culture treated polystyrene (PS) surface as control as it has been previously reported in the literature and was used as a substrate for deposition of our hydrocolloidal films [11,49]. The results obtained here agreed with the findings previously described for this surface, which displays characteristic fiber-like topographical features (Figure 5A) [11,49]. The rest of the surfaces (Figure 5A) displayed a mix of globular and fiber-like features. The globular appearance of the films was not unexpected since egg white is composed of globular proteins [18]. Regarding the measured Ra (Figure 5B), only the 1% EW surface had a higher Ra than the PS control surface. This would suggest that the protein hydrocolloids deposit filling the voids and thus rendering a more homogeneous and flat surface. For the 1% EW hydrocolloid, the amount of protein is not enough to fill the voids; therefore, the protein is deposited randomly throughout the surface, thereby creating additional globular deposits that increase Ra. Finally, the values obtained here for PS and 1% Gel surfaces were very similar to those reported by Frost et al. [11], indicating the reproducibility and simplicity of the coating method used for producing hydrocolloid films.

### 2.3. Two-Dimensional Hydrocolloid Films of Egg White and Gelatin: Cell Morphology, Viability, and Proliferation

**Figure 6:**
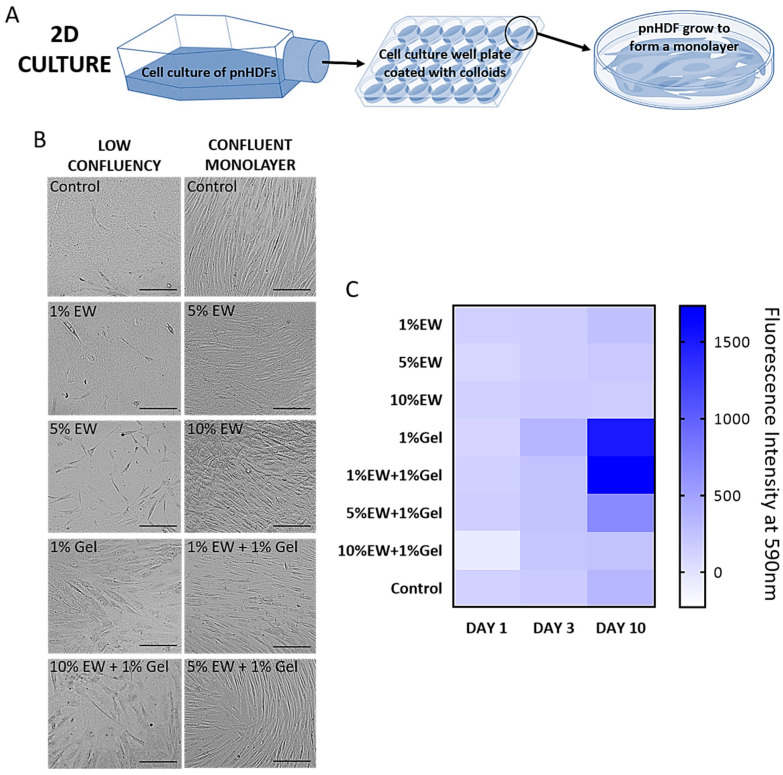
(**A**) Scheme showing 2D culture of primary normal human dermal fibroblasts (pnHDFs) on cell culture well plates coated with hydrocolloid films. (**B**) Representative phase contrast light microscopy of pnHDF on hydrocolloid films at low confluency (**left**) and as a confluent monolayer (**right**). Scale bar = 100 µm. (**C**) Heat map of cell viability and proliferation (alamarBlue^®^ assay) compared with the control showing mean of N = 3 per hydrocolloid type. Control is uncoated tissue-culture treated polystyrene (PS) wells.

Primary normal dermal fibroblasts (pnHDF) were cultured on the hydrocolloid films (Figure 6A) and their cell morphology, viability, and proliferation studied. Fibroblasts were chosen due to their ubiquitousness in soft connective tissues, as well as being responsible for depositing the ECM characteristic of these tissues [50].

For cell morphology, it was monitored whether cells kept their typical shape and were able to eventually form a monolayer. Typically, fibroblast morphology is elongated. One elliptical nucleus with multiple nucleoli can be observed in these cells as well as a prominent endoplasmic reticulum. At high confluence, fibroblasts form a confluent monolayer with parallel clusters [51]. Changes in cell morphology in response to biomaterial surface, which are easily observable by phase contrast light microscopy, are indicative of possible toxic effects, thereby inducing processes such as necrosis or apoptosis, or more complex responses such as senescence [52,53,54]. In this study, cells grew over time eventually forming a confluent monolayer on all surfaces (Figure 6B). On the control wells, cells displayed the typical fibroblast morphology and confluent monolayers showed the parallel clusters described in the literature (Figure 6B). In the wells coated with the hydrocolloid solutions cells kept the typical fibroblast features and multiplied over time, eventually yielding a confluent monolayer. Parallel clusters were also observed, although they were not as regular as the ones formed on the control surfaces (Figure 6B: as an example, note the difference in morphology between the control monolayer and the 10% EW monolayer), due to the more globular morphology of the hydrocolloidal coatings compared with the fiber-like topological features of the polystyrene control wells (Figure 5A).

Plotting a heat map (Figure 6C) allowed us to screen the cell viability and growth on the different hydrocolloids compared with the control (uncoated well), with main differences in cell growth observed at day 10 of culture. Cells remained viable and proliferated on all the hydrocolloid films as well as on the control wells; 1% Gel, 1% EW + 1% Gel, and 5% EW + 1% Gel films had the highest increase in cell proliferation values.

Cells cultured on 1% gelatin showed increased proliferation compared with the control, which was expected as this coating is often used to maximize cell culture [51]. Gelatin exposes short aminoacid sequences, i.e., Arg-Gly-Asp (RGD), recognized by integrins thereby promoting cell attachment [51]. EW proteins lack these attachment motifs that are introduced by combining EW with gelatin into the mixed hydrocolloids, which showed a clear trend: the higher the percentage of EW, the lower the proliferation probably due to dilution of RGD sequences.

### 2.4. Miniaturized 3D Environments of Egg White and Gelatin

**Figure 7:**
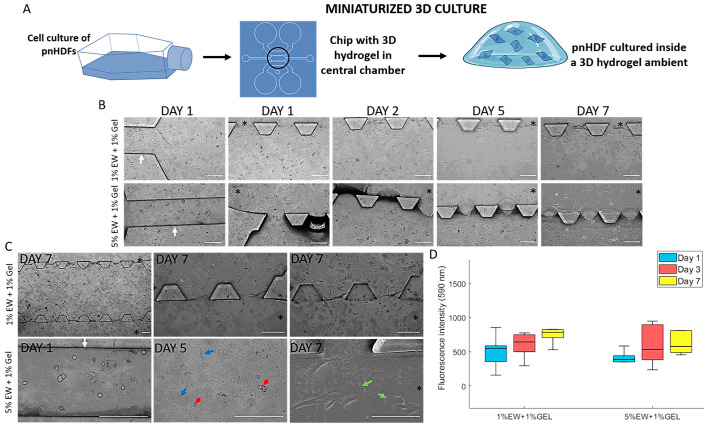
(**A**) Scheme showing miniaturized 3D culture of primary normal human dermal fibroblasts (pnHDFs) inside microfluidic devices loaded with EW and gelatin hydrocolloids. (**B**,**C**) Phase contrast light microscopy of pnHDF inside microfluidic devices. Scale bar is 175 µm. * Indicates large hydrating channels while white arrows point to small channels. (**B**) Representative images showing cell morphology over time. (**C**) Additional representative images. For the 1% EW + 1% Gel panel, the last 2 photos are the exact same view at different planes of observation, showing cells on both planes. Blue arrows point at examples of cells with a flat morphology whilst red arrows point at rounded cells. Green arrows show lamellipodia. (**D**) alamarBlue^®^ assay results showing median (central line), interquartile range (box), and minimum and maximum values (whiskers) of N = 5 per hydrocolloid type. No statistical differences were found.

Microfluidic devices have gained notoriety in the last decade due to their ability to create miniaturized controlled environments for TERM experimentation. Perhaps the main advantage of these systems is their miniature size, which requires a minimum amount of materials, reagents, and cell numbers, making them very cost-effective. Importantly, 3D environments, co-cultures, and spheroid and organoid culture can be achieved inside them, which allows the creation of tissue or organ-like structures, thereby gaining the popular name of organs-on-a-chip [55]. These miniaturized devices are being used for a variety of tissue engineering purposes, from cancer-on-a-chip models to bone regeneration [56,57]. In this work, we hypothesized that the hydrocolloids of egg white and gelatin can be used to create miniaturized 3D hydrogel environments inside microfluidic devices (Figure 7A). Based on the screening results on 2D surfaces, the hydrocolloids 1% EW + 1% Gel and 5% EW + 1% Gel were used for this purpose.

The hydrocolloids were easily introduced and confined into the central chamber. Contrast light microscopy showed that cells introduced through the small channels migrated to the central chamber and over time, to the large hydration channels (Figure 7B,C). For the devices loaded with 1% EW + 1% Gel, both flat and rounded cells can be seen in the small channels and the central chambers on day 1 (Figure 7B). Over time, mostly flat cells could be observed, indicating a predominantly flat substrate. Nevertheless, cells can be seen on different planes on day 7 (Figure 7C), indicating a degree of three-dimensionality. For the 5% EW + 1% Gel-loaded devices, mostly rounded cells, can be seen, as expected in a 3D matrix (Figure 7B). Over time, flat cells can also be seen, and they appeared embedded in the 3D matrix. Cells that migrated out into the large hydrating channels were flat and showed a migrating morphology, with clearly visible lamellipodia (Figure 7C, green arrows). Cell growth was observed to be slightly higher in the 1% EW + 1% Gel hydrogel compared with 5% EW + 1% Gel, although this difference was not significant (Figure 7D).

Results on the microfluidic devices suggested that hydrocolloids of EW and gelatin can be used for cell studies using these miniaturized cell culture platforms. Interestingly, a 3D environment can be created inside the devices, especially with the 5% EW + 1% Gel hydrocolloid, which adds biomimicry to these systems as cells’ natural environment is a 3D ECM [2]. Further work using these hydrocolloids in microfluidic devices involves the study of cellular behavior such as cell migration and matrix remodeling.

### 2.5. Three-Dimensional Hydrogel Scaffolds of Egg White and Gelatin: Morphology and Architecture

The last decade has seen the rise of hydrogels as biomaterials of choice for TERM applications, as they possess a range of properties that make them very attractive for tissue interaction and regeneration. Hydrogels present a hydrated and viscoelastic nature, which provides an ideal environment for cell survival and function and simulate the native ECM in terms of structure [21,58]. The last part of our study focused on fabricating 3D hydrogel scaffolds and characterizing their material and cell properties (Figure 2D).

**Figure 8:**
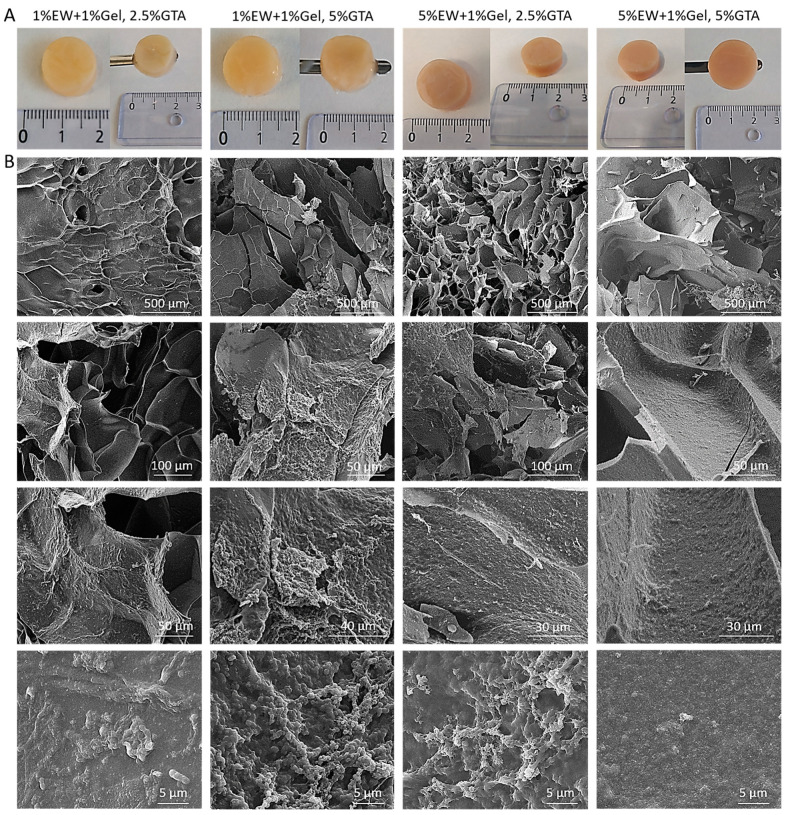
(**A**) Macroscopic appearance of freshly fabricated 3D hydrogel scaffolds before lyophilization. (**B**) Representative SEM images of lyophilized 3D hydrogel scaffolds. Images at the bottom (scale bar = 5 µm) show nano-structures. EW: egg white; Gel: gelatin; and GTA: glutaraldehyde.

We used a fabrication method with sequential temperature-dependent gelation followed by chemical cross-linking of the polymeric network of the hydrogel for added mechanical strength and stability (see Section 4.6 and Figure 2D for detailed explanation of our fabrication method). We fabricated four different prototypes with varying EW quantities and crosslinker’s (glutaraldehyde (GTA)) concentration. Once removed from the mold, 3D hydrogel scaffolds were obtained using 1% EW + 1% gelatin and 5% EW + 1% gelatin hydrocolloids (Figure 8A). It was observed that all prototypes had a marble-like and consistent appearance and were yellowish/orangish in color depending on the glutaraldehyde concentration used for the cross-linking and the amount of EW in the gel, being darker at higher concentrations of both EW and glutaraldehyde. All prototypes were easily handled with a spatula (Figure 8A).

Images obtained under SEM (Figure 8B) showed hydrogel scaffolds formed organized and well-defined internal structures. The scaffolds displayed pores as well as lamellar, flat structures. Materials with lamellar structures are the object of study and interest in tissue engineering and biomaterial science since several biological structures present lamellae. Examples are blood vessels, which display concentric layers of elastic lamellae, or lamellar bone [59,60,61]. At high magnifications, a globular nano-topography as well as nano-pores, were observed, which is important for maximizing cell interaction as cells in our bodies are in contact with nano-features [62]. The observed architecture also explains why flat, elongated cells were observed in the microfluidic devices: as cells rest on the lamellae walls, they adopt a flat and elongated morphology. 

### 2.6. Three-Dimensional Hydrogel Scaffolds of Egg White and Gelatin: Rheology and Swelling

Rheology and swelling investigation allowed us to obtain information about the viscoelastic properties of the scaffolds as well as of their 3D polymeric network.

**Figure 9:**
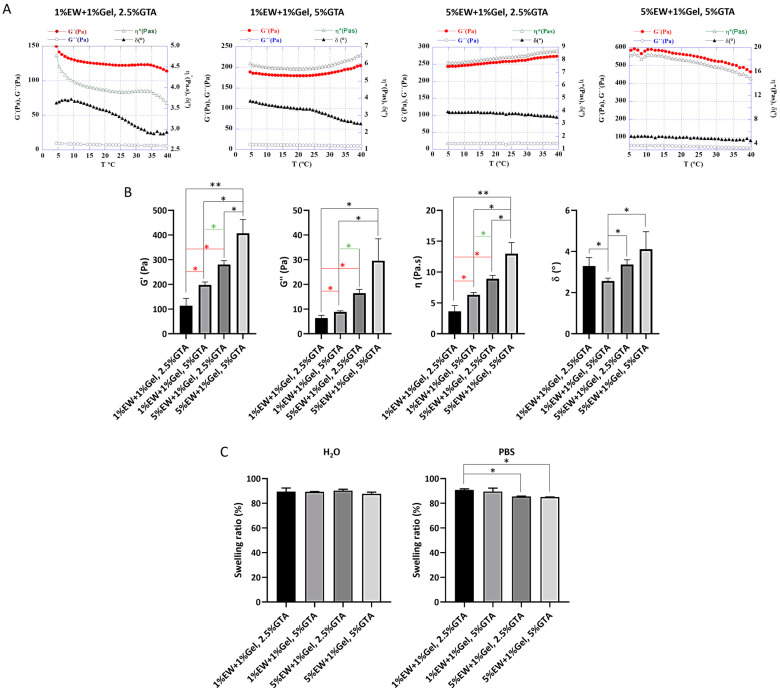
(**A**) Representative rheological profiles of the 3D hydrogel scaffolds. Graphs show the storage G’ and loss modulus G” (red and blue respectively, measured in Pa), phase angle δ (black, measured in °), and specific viscosity η* (green, measured in Pa.s) of each sample recorded against increasing temperature (T, measured in °C). (**B**) G’, G”, η*, and δ values at 37 °C of the 3D hydrogel scaffolds (mean ± SD of at least N = 3). (**C**) Swelling ratio of the 3D hydrogel scaffolds in both H_2_O and PBS (mean ± SD of an N = 2) after 15 min at 37 °C. * *p* < 0.05 and ** *p* < 0.001.

Rheology results showed that the 3D hydrogels were stable over the experimental temperature range as G’, G”, η*, and δ displayed similar values from 5 to 40 °C (Figure 9A). These results suggest that the fabricated scaffolds would be stable in terms of viscoelastic properties at handling and physiological temperatures. G’ was higher than G” indicating a predominantly elastic behavior over a viscous one [63,64]. Furthermore, G’ was observed to be significantly higher as the percentage of protein (EW) and crosslinker increased (Figure 9A,B), indicating that both protein and crosslinker concentration play a role in the resulting material’s rigidity [64,65]. For instance, it has been shown that the larger the amount of ovalbumin, the higher number of disulphide bonds are formed during gelation [66]. Therefore, the mechanical properties of 3D hydrogels of egg white and gelatin can be tailored by modifying the amount of protein and/crosslinker concentration, making these materials attractive for tissue engineering studies where controlled modification of matrix stiffness is of paramount importance. In terms of values, the 3D hydrogels presented here obtained higher G’ and G” than those reported for collagen gels, the most utilized hydrogels in tissue engineering applications. For instance, Valero and colleagues reported G’ values ranging from 13.35 ± 0.36 Pa (1.5 mg/mL collagen gel) to 258.05 ± 3.89 Pa (crosslinked 6.0 mg/mL collagen gel) [65], compared with G’ values shown here ranging from 113.80 ± 29.85 Pa (1% EW + 1% Gel, 2.5% GTA prototype) to 407.10 ± 55.7 Pa (5% EW + 1% Gel, 5%GTA prototype). Therefore, the 3D hydrogels of egg white and gelatin can be used in applications where the hydrogel matrix needs increased stiffness.

Water interaction with synthetic and natural hydrogels is of elemental importance in biomaterial science since water is the most abundant and fundamental component of biological systems [67]. Interaction of materials with water is key in determining the physiochemical and biological responses. The hydrophilic polymer that constitutes a hydrogel’s 3D network can swell in and keep a significant volume of water whilst maintaining its architecture due to physical or chemical crosslinking of the macromolecular chains. The degree of swelling specifies an interaction between the scaffolds and the aqueous medium, and it is the consequence of solvent trying to dilute the scaffolds polymeric network by penetration [68]. Scaffolds were placed in water and PBS as aqueous solvents that were absorbed by the polymeric network subsequently causing swelling of the scaffolds, which is directly proportional to the degree of crosslinking, as swelling decreases with the degree of crosslinking (Figure 9C), in agreement with the literature [69]. Between 85 and 90% swelling was observed in both water and PBS. Swelling in PBS decreased for the scaffolds with a higher protein content (5% EW + 1% Gel). This can be attributed to the higher degree of crosslinking because of the increased amount of protein in the network, as well as the increased ionic strength of PBS compared with water, as the presence of ions would be expected to affect the equilibrium swelling of the hydrogels [70]. In summary, our results indicate the high affinity of the fabricated 3D hydrogels for water.

### 2.7. Three-Dimensional Hydrogel Scaffolds of Egg White and Gelatin: Cell Proliferation and Penetration

**Figure 10:**
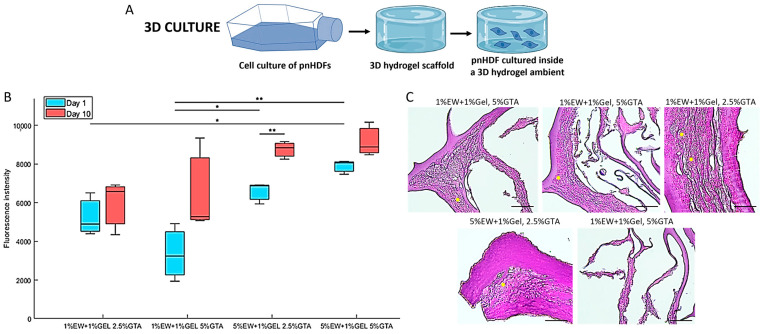
(**A**) Scheme of 3D culture of primary normal human dermal fibroblasts (pnHDFs) in 3D hydrogel scaffolds. (**B**) alamarBlue^®^ assay results showing median (central line), interquartile range (box), and minimum and maximum values (whiskers) of N = 3 per 3D hydrogel scaffold. * *p* < 0.05 and ** *p* < 0.001. (**C**) Representative H and E-stained sections of seeded 3D hydrogel scaffolds at day 10 of culture (dark pink = matrix, purple dots = cell nuclei). Yellow dots indicate pores between consecutive lamellae filled with cell layers. Scale bar = 50 µm.

When cells were cultured in 3D hydrogel scaffolds (Figure 10A), results showed that cells were able to attach, remain viable, and proliferate in the hydrogel scaffolds (Figure 10B,C). The prototypes 5% EW + 1% Gel showed the highest cell proliferation (Figure 10B), which may be due to the increased mechanical properties of these hydrogels compared with the 1% EW + 1% Gel prototypes, as fibroblasts proliferation has been found to increase with increased matrix stiffness [71]. In terms of cell penetration, cells were seen to colonize the scaffolds and grow in layers on the lamellae walls, even filling the pores between consecutive lamellae walls (Figure 10B, yellow dots). Nevertheless, we acknowledge that the eosin staining of the scaffold matrix was very strong, probably due to the high protein content of the scaffolds, and slightly interfered with cell nuclei observation, pointing out the need for future improvement of cell observation inside these scaffolds. Finally, Figure 10C confirms the lamellar nature of the scaffolds fabricated here, displaying both parallel and perpendicular lamellae of varying thicknesses.

## 3. Conclusions

This paper presents a platform for hydrogel-based tissue engineering combining hydrocolloids of egg white and gelatin, which have been investigated in the food chemistry field but not for TERM applications. Here we introduce the application of these mixed hydrocolloids to biomedicine. This platform allows the fabrication of thin 2D hydrocolloid films with globular nano-topography, miniaturized 3D hydrogels inside microfluidic devices, and 3D hydrogel scaffolds with pores, lamellae, and globular nano-topography with tunable mechanical properties. The hydrocolloids investigated here present low-production costs and easy availability compared with other natural materials such as collagen or fibrin, and their temperature-dependent properties as well as tunable mechanical properties can be exploited for producing a range of substrates and 3D hydrogel materials. The results presented in this paper warrant future investigation of these hydrocolloids for various and different TERM areas, including their use in cancer models, organoid growth, implantable medical devices, or compatibility with bioprinting techniques. Therefore, the large range of properties and characteristics of these materials provide a strong potential for a large variety of medical applications.

## 4. Materials and Methods

### 4.1. Experimental Design Summary

Our experimental design consisted of four differentiated blocks (Figure 2). In the first block, we focused on the hydrocolloid solutions, their preparation, appearance, and temperature-dependent gelation properties, which were investigated by rheology (Figure 2A). The second block addressed the use of the EW and gelatin hydrocolloids as thin coating films, i.e., 2D substrates for cell culture. Hydrocolloid films were characterized by atomic force microscopy (AFM), and their effect on cell viability, proliferation, and morphology were assessed (Figure 2B). The third experimental block investigated the use of EW and gelatin hydrocolloids to create miniaturized 3D hydrogels inside microfluidic devices. Cell viability, proliferation, and morphology were assessed in these microfluidic devices loaded with 3D hydrogels of EW and gelatin (Figure 2C). Finally, the fourth block focused on 3D hydrogel scaffolds of EW and gelatin and their fabrication, material characterization, and cellular properties (viability, proliferation, and penetration) (Figure 2D).

### 4.2. Egg White and Gelatin Hydrocolloids

Large chicken (*Gallus gallus domesticus*) eggs from a local farm (Zaragoza, Aragon, Spain) were cracked open and their contents placed on Petri dishes. Avoiding the yolk and chalaza, the egg white (EW) was transferred to a Falcon tube. The EW was then lyophilized and a yellowish powder of about the same volume as the solution was obtained. The lyophilized EW powder was kept at 4 °C until used.

Egg white (EW) and gelatin (Gel, type B from bovine skin, Merck, Madrid, Spain) colloidal solutions prepared in dH_2_O are detailed in Table 1. The pH of the hydrocolloids was adjusted to pH = 7.0 ± 0.5 (physiological pH) to prevent cytotoxicity, since these solutions were used for producing 2D coating films and 3D hydrogels that underwent cell seeding and culture. The solutions were kept at 4 °C (up to 3 days) until used.

### 4.3. Rheology of Hydrocolloids

The hydrocolloid solutions from Table 1 were characterized by rheological assays using a stress-controlled rotational rheometer HAAKE Rheostress 1 (Thermo Fisher Scientific, Waltham, MA, USA). All samples were tested using both a cone-plate and a plate-plate configuration with a 35 mm diameter and a cone angle of 1°. With the cone-plate sensor, 500 μL of each sample were pipetted on the lower plate of the rheometer at 5 °C, whilst it was 1000 μL in the case of the plate-plate sensor. Then, the upper cone or plate descended until the gap between both plates was the required by the sensor specifications (0.051 mm for cone-plate and 1 mm for plate-plate). A solvent trap was used to avoid sample dehydration. After allowing the sample to stabilize for 5 min at 5 °C, the shear test was executed applying a fixed torque of 5 µNm at the frequency oscillatory of 1 or 5 Hz, both within the linear viscoelastic region (LVR) with a strain of amplitude of 0.5% or 1%, respectively. The storage G’ and loss modulus G”, phase angle δ, and specific viscosity η* of each sample were recorded while the temperature gradually increased from 5 to 70 °C at a constant heating rate.

### 4.4. Coating of Cell Culture Well Plates

Cell culture well plates (12 and 24 wells, VWR, Radnor, PA, USA) were coated with the different colloidal solutions (Table 1). Enough solution to cover the bottom of the well was added and the plates left overnight at 4 °C (Figure 2B). The excess solution was then removed, and the plates washed with 70% ethanol to sterilize the surface prior to cell seeding. This was followed by three washes with phosphate buffered saline (PBS) before cell seeding.

### 4.5. Microfluidic Platform

Microfluidic devices used were previously described and are routinely used by our group [56,72,73,74]. Briefly, we used soft lithography to develop positive SU8 240-µm relief patterns with the desired geometry on a silicon wafer (Stanford University). Polydimethylsiloxane (PDMS, Sylargd 184, Dow Corning GmbH, Wiesbaden, Germany) was mixed at a 10:1 weight ratio of base to curing agent, the solution poured into the SU8 master and degassed to remove air bubbles. The replica-molded layer was then trimmed, perforated, and autoclaved. Finally, the PDMS device and 35 mm glass-bottom petri dishes (Ibidi, Gräfelfing, Germany) were plasma-bonded.

The device geometry (Figure 2C) contained a central chamber into which the sterile-filtered hydrocolloids were deposited through the small channels (Figure 2C). Gelation occurred by heating the devices at 80 °C for 30 min followed by cooling at 4 °C overnight, thereby yielding a hydrogel. The chip was washed once with PBS before loading the cell suspension into the central chamber containing the hydrogel through the small channels. Two large side media channels running parallel and connected to the central chamber ensured hydration and transport of nutrients (Figure 2C).

### 4.6. Three-Dimensional Hydrogel Scaffolds

Three-dimensional hydrogel scaffolds were fabricated to ~14 mm diameter and ~4 mm thickness. Once poured onto the hydrophobic mold, the hydrocolloids were heated at 80 °C for 2 h followed by cooling at 4 °C overnight. The resulting gels were chemically cross-linked with either 2.5% or 5% glutaraldehyde (Merck, Madrid, Spain) for 4.5 h at room temperature, followed by 48 h at 4 °C. Scaffolds were thoroughly washed with dH_2_O and lyophilized (Figure 2D).

### 4.7. Atomic Force Microscopy (AFM)

Cell culture treated polystyrene (PS) surfaces of ~1 cm^2^ were coated with the different hydrocolloids as explained in Section 2.4. Field images of 50 × 50 µm and areas of 3 × 3 µm were scanned by a MultiMode V atomic force microscope (AFM) (with Nanoscope V Controller, Bruker, Santa Barbara, CA, USA) using tapping mode (TM) in air to assess the topography morphology and characterize the roughness corresponding to eight tested samples, respectively. TM vibrates the AFM microlever near its resonance frequency controlling the tip–sample interaction by the oscillation damping. This aspect favors the significant reduction in detrimental lateral forces, rendering straightforward non-destructive imaging data acquisition, allowing the surface morphological characterization with sub-nanometer resolution [75]. AFM images were taken using rectangular silicon TESPA-V2 probes (Bruker, Santa Barbara, CA, USA) with nominal resonant frequency of 320 KHz. The nominal sharp apex radius was 7 nm minimizing non-desirable tip convolution broadening effects [76]. The AFM images were recorded at a scanning rate of 0.8 Hz with a resolution of 512 pixels/line. Roughness arithmetic average (Ra) parameter of the absolute values of the sample surface height deviations measured from the mean plane was estimated (Equation (1)), where *N* is the population size, *Z* is the absolute height value, and *j* is the number of the specific local image area taken into consideration, respectively.

(1) $$R_{a}=\frac{1}{N}\sum_{i=1}^{N}\left|Z_{j}\right|$$

Three different areas (N = 3) of 3 µm × 3 µm were analyzed to estimate Ra for each tested surface sample. Raw AFM images were processed by Gwyddion [77], WSxM [78], and Nanoscope Analysis software tools. All AFM images were analyzed following the same procedure and settings in order not to induce Ra deviations based on data handling.

### 4.8. Scanning Electron Microscopy (SEM)

Lyophilized 3D hydrogel samples were coated with a carbon film. An InspectTM F50 SEM (FEI Company, Hillsboro, OR, USA) in an energy range between 0 and 30 keV was used to acquire SEM images of the 3D hydrogel scaffolds.

### 4.9. Swelling Ratio of 3D Hydrogel Scaffolds

The swelling ratio of 3D hydrogel scaffolds was calculated with Equation (2):

(2) $$SR=\frac{M_{w}-M_{d}}{M_{w}}\cdot 100$$

where *M_d_* is the dry mass and *M_w_* is the wet mass of the scaffold. Lyophilized scaffolds were cut to 5 × 5 mm square pieces and weighted (time 0). Wet mass was calculated by submersing the scaffold into 5 mL of distilled water or phosphate buffered saline (pH = 7.4) for 15 min at 37 °C. Fifteen minutes was sufficient to reach a ready state of swelling that did not change with longer submersion times [79].

### 4.10. Rheology of 3D Hydrogel Scaffolds

The 3D hydrogel scaffolds, which were prepared fresh and were measured within 2–3 days of preparation, were characterized by rheology (HAAKE Rheostress 1, Thermo Fisher Scientific, Waltham, MA, USA) using a plate-plate configuration with a 35 mm diameter. Scaffolds were cut to ~14 mm diameter and ~2 mm thickness and placed on the lower plate of the rheometer at 5 °C. Then, the upper plate descended, touching the scaffold. After allowing the sample to stabilize for 5 min at 5 °C, the shear test was executed applying a fixed torque of 5 µNm at the frequency oscillatory of 5 Hz within the linear viscoelastic region (LVR). The storage G’ and loss modulus G”, phase angle δ, and specific viscosity η* of each sample were recorded while the temperature gradually increased from 5 to 40 °C at a constant heating rate.

### 4.11. Cell Culture

Primary normal human dermal fibroblasts (pnHDF, Lonza, Basel, Switzerland) were cultured in Dulbecco’s modified Eagle’s medium (DMEM, Gibco, Madrid, Spain) supplemented with 10% fetal bovine serum and 1% antibiotic/antimycotic solution. The medium was changed every 3 days. Cells were regularly observed under a phase contrast light microscope and photographed. Cells were used between passages 5 and 12, when they were proliferative.

### 4.12. Cell Seeding

On 2D surfaces, 1000 cells/well for cell viability and proliferation and 10,000 cells/well for cell morphology observation were seeded. About 500 cells/microfluidic devices were seeded for both cell viability and proliferation and cell morphology observation. A total of 20,000 cells/3D hydrogel scaffold (5 mm × 5 mm square pieces) were seeded for both cell viability and proliferation and cell penetration into the 3D scaffolds.

### 4.13. Cell Viability and Proliferation by AlamarBlue^®^ Assay

Cell proliferation was assessed by the metabolic redox assay alamarBlue^®^. On 2D surfaces (N = 3 per surface), the medium was removed from each well and cells were washed with PBS before adding 1 mL of alamarBlue working solution (1/10 alamarBlue^®^, made up in DMEM, DAL1025, Invitrogen, Madrid, Spain) and incubated for 2 h at 37 °C, 5% CO_2_. The 1 mL samples were transferred to a 96 well plate by adding 100 µL of sample in each well. Triplicate measures of the samples were made.

For microfluidic devices (N = 5 per hydrocolloid type), 200 µL of alamarBlue working solution were added per chip, which were then incubated for 4 h at 37 °C, 5% CO_2_. The 200 µL samples were transferred to a 96 well plate, by adding 100 µL of samples in each well. Duplicate measures of each sample were made.

For 3D hydrogel scaffolds (N = 3 per scaffold prototype), 1 mL of alamarBlue working solution (made up in PBS) was added per scaffold and incubated for 4 h at 37 °C, 5% CO_2_. The 1 mL samples were transferred to a 96 well plate, by adding 100 µL of sample in each well. Triplicate measures of the samples were made.

Fluorescence intensity was read (excitation at 530 nm and emission at 590 nm) using a plate reader (Synergy LX, BioTek with Gen5 3.10 software).

### 4.14. Phase Contrast Light Microscopy

Morphology of cells in the different cultures, i.e., 2D coated surfaces (N = 3 per surface) and 3D cell culture in microfluidic devices (N = 5 per hydrocolloid type), was observed by phase contrast light microscopy (DM IL LED, Leica, Wetzlar, Germany) and photographed.

### 4.15. Cell Penetration into 3D Hydrogel Scaffolds

Seeded scaffolds (N = 3 per prototype) on day 10 of culture were fixed in 4% paraformaldehyde, processed for paraffin histology, and cut into sections that were stained with Haematoxylin and Eosin (H and E). Stained sections were imaged and photographed (Leica DMi1, Wetzlar, Germany).

### 4.16. Data and Statistical Analysis

The following software packages were used: Microsoft Excel 365, GraphPad Prism 8.0.1, OriginPro 8.5, KaleidaGraph 3.6, and Matlab R2022a. One-way analysis of variance (One-way ANOVA) and Tukey’s test statistical calculations were carried out for the Ra values of the assessed sample surfaces. For the alamarBlue^®^ data, comparisons between groups were made using one-way ANOVA with a Tukey–Kramer post-hoc analysis. For swelling ratio and rheology results, an unpaired t-test was performed. A *p*-value below 0.05 was considered a significant result.

## Figures and Tables

**Table 1:** Colloidal solutions prepared in this study. For exact color evaluation, please see Figure 3.

Hydrocolloid (% in *w*/*v*)	Observations
1% Gel	Liquid, yellowish in color, and translucid
1% EW	Liquid, yellowish in color, and translucid
5% EW	Viscosity in between 1% and 10% egg white colloids, yellowish in color, and translucid
10% EW	Thick, viscous, yellowish in color, and translucid
1% EW + 1% Gel	No observable differences compared with 1% egg white colloid
5% EW + 1% Gel	No observable differences compared with 5% egg white colloid
10% EW + 1% Gel	No observable differences compared with 10% egg white colloid

## Data Availability

Data will be made available on request.
